# Development and testing of a precision hoeing system for re-compacted ridge tillage in maize

**DOI:** 10.1016/j.heliyon.2024.e40527

**Published:** 2024-11-20

**Authors:** Oyebanji O. Alagbo, Marcus Saile, Michael Spaeth, Matthias Schumacher, Roland Gerhards

**Affiliations:** a Department of Weed Science (360b), Institute of Phytomedicine, University of Hohenheim, 70599, Stuttgart-Hohenheim, Germany; b Department of Crop Production and Protection, Faculty of Agriculture, Obafemi Awolowo Univeristy, Ile-Ife, Nigeria

**Keywords:** Ridge tillage, Flat tillage, Soil compaction, Mechanical weed control, Herbicide

## Abstract

Ridge tillage (RT) is a conservation practice that provides several benefits such as enhanced root growth and reduced soil erosion. The objectives of this study were to develop an autosteered living mulch seeder and hoeing prototype for RT systems using RTK-GNSS (real-time kinematic global navigation satellite systems) created ridges as a guide. It was also aimed to compare weed control efficacy and crop response of ridge-hoeing compared to conventional hoeing in flat tillage (FT). It was further aimed to investigate the impact of a new RT technology (with ridge re-compaction) on maize root development, yield, soil temperature, and moisture compared to FT.

Field experiments were conducted with maize in 2021 and 2022 in a two-factorial split-plot design with tillage (RT and FT) as main treatment and weed control (untreated, herbicide, twice hoeing, hoeing + living mulch) as sub-treatment factors. Weed density, coverage, biomass, crop density, weed control efficacy (WCE) and maize silage yield were assessed. Temperature loggers were installed within RT and FT to take temperature readings at 20 min. Soil moisture and root penetrability were measured every two weeks in each plot using soil samples and a penetrometer.

The WCE and yield did not differ significantly between the tillage systems. Twice hoeing resulted in 71–80 % WCE, which was equal to herbicide treatment. Hoeing + living mulch achieved 70–72 % WCE. Different from previous studies with ridge tillage, temperatures in the compacted ridges did not consistently differ from the ridge valleys and flat seedbeds. Root penetration (against 1.4 MPa penetrometer cone index) was 40 % higher in RT than in FT. On average, RT maize produced more (53.6 g m^−2^) root biomass compared to FT. In summary, re-compacted ridges built along RTK-GNSS lines can allow post-emergent hoeing and living mulch seeding along ridges and also provide good growing conditions for maize.

## 1 Introduction

Ridge tillage (RT) represents a tool in Conservation Agriculture aiming to reduce energy consumption, soil erosion, and nitrogen leaching while also improving soil physicochemical properties [1]. RT provides better growing conditions for the crop on the top of ridges due to higher soil temperatures, lower soil penetration resistance, and enhanced nutrient mineralization [[2], [3], [4],^5^]. RT often results in higher yields compared to conventional flat seedbeds [6,2], especially under very dry and very wet field conditions [[7], [8], [9], [10]]. Higher soil temperature provided by ridges can induce faster germination and emergence of crops [6,2,7,8,11]. Moreso, unlike flat seedbeds, ridges provide a large surface area, with lower root penetration resistance – suggesting better root aeration [3,12,13].

Moreover, the favourable soil microclimate provided by RT could equally promote dominance of problem weeds and their accumulation in soil seed bank, due to less intensive tillage practices compared to flat tillage systems [14,15]. For instance, the soil seed bank of permanent RT contains a high number of dicotyledonous species such as *L. purpureum*, *S. media*, and *Amaranthus* spp. as well as warm season grasses like *Digitaria sanguinalis* L. (15 16). Herbicide resistance issues have been reported in RT due to the heavy reliance of herbicides for weed control [16,17]. In organic RT systems, manual weeding is cost-intensive [15]. Hence, precise, low-cost, and environment-friendly weed control options for RT systems are needed. The European Union directives for Integrated Pest Management [18] recommends the combination of preventive and curative methods of weed control to reduce the reliance on herbicides, avoid their negative environmental impacts, and prevent the evolution of herbicide resistance in weed populations [19]. At the early growth stage of the crop, optimal growth conditions provided for root development in RT could enhance crop competitiveness against weeds and may further improve the tolerance of the crop to mechanical weeding [20]. Furthermore, RT can combine mechanical weeding (on ridges) with living mulches (in valleys) to suppress weeds. Similarly, herbicides can also be sprayed in a band (on the top of ridges) in combination with mechanical weeding [20].

Conventional ridgers such as potato ridge cultivators [14], as well as single, double, or multiple-pair disc ridgers [21], and rotary tillers have been used for ridge cultivation in cereals, tuber crops, legumes, and vegetable fields all around the world with better yield outcomes [13,15,21,5] compared to flat seedbeds. Alagbo et al. [20] introduced a new RT system, where ridges are built with RTK-GNSS followed by ridge re-compaction with heavy prism rollers (*Glühfosator*, Frost, Germany). The new technology allows for autosteered sowing and precise post-emergent hoeing taking ridge furrows as a guide. Re-compaction is expected to improve the movement of water capillaries into the ridge areas thereby optimizing the benefit of ridges to crops. However, whether growing conditions for the crops can be improved by re-compacting the ridges is not known. If the ridges were created with RTK-GNSS guided machinery and the crop was sown exactly on the top of the ridge, those ridges could be used for precise hoeing closely along the crop rows on the ridges, as well as for living mulch sowing in the valleys. The objectives of this study were to develop autosteered hoeing prototypes and living mulch seeding elements for RT systems using RTK-GNSS-created ridges as a guide. It also aimed to compare the weed control efficacy, crop response, and yield of tested ridge-hoeing prototypes with conventional weed control in flat seedbeds. Finally, it aimed to investigate the impact of ridge re-compaction on maize root development, soil temperature, and moisture compared to conventional flat tillage (FT).

## 2 Materials and methods

### 2.1 Development of ridge hoe and living mulch seeder

**Fig. 1:**
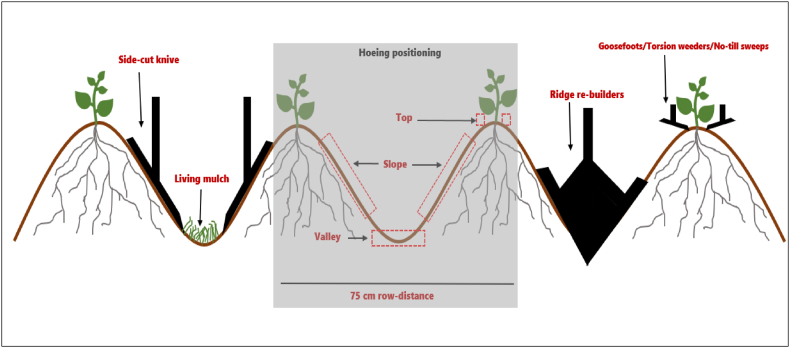
Schematic description of weed control in ridge tillage with crops on the top of the ridges, living mulch in ridge valleys and positioning of hoeing elements on top (torsion weeders, goosefoots or no-till sweeps), slopes (down side-cut knives), and valleys (ridge re-builders) of ridges [20].

**Fig. 2:**
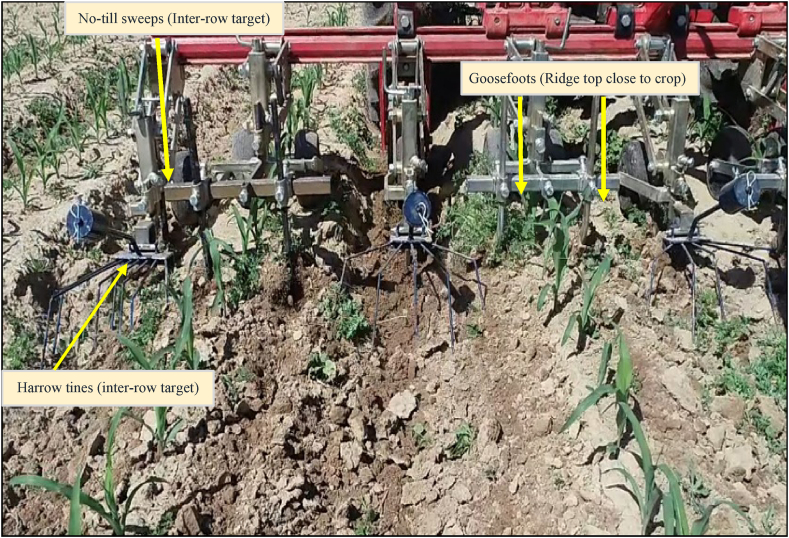
Ridge hoeing arrangement on a 3-m hoeing frame in 2021. On ridge tops (intra row target), pairs of goosefoot sweeps (9 cm wide) were positioned 5 cm away from maize plants. In the valleys and slopes of ridges (inter-row target), no-till sweeps (20 cm wide) were mounted followed by harrowing tines.

A preliminary study began in 2021 to test and select preferred ridge hoeing arrangements on a 3-m wide hoeing frame [20]. Different hoeing options on the ridge tops, slopes, and valleys were modelled, including living mulch seeding in the valley area of ridges (Fig. 1). In 2021, pairs of goosefoot sweeps (9 cm wide) were mounted 5 cm next to the maize plants (left and right) to remove weeds from the top of the ridges (intra-row target). No-till sweeps (20 cm wide) were mounted and positioned precisely within ridge valleys, followed by harrow tines mounted behind each no-till sweep (inter-row target) – to lift the soil, uproot, and expose weeds to sunlight (Fig. 2).

**Fig. 3:**
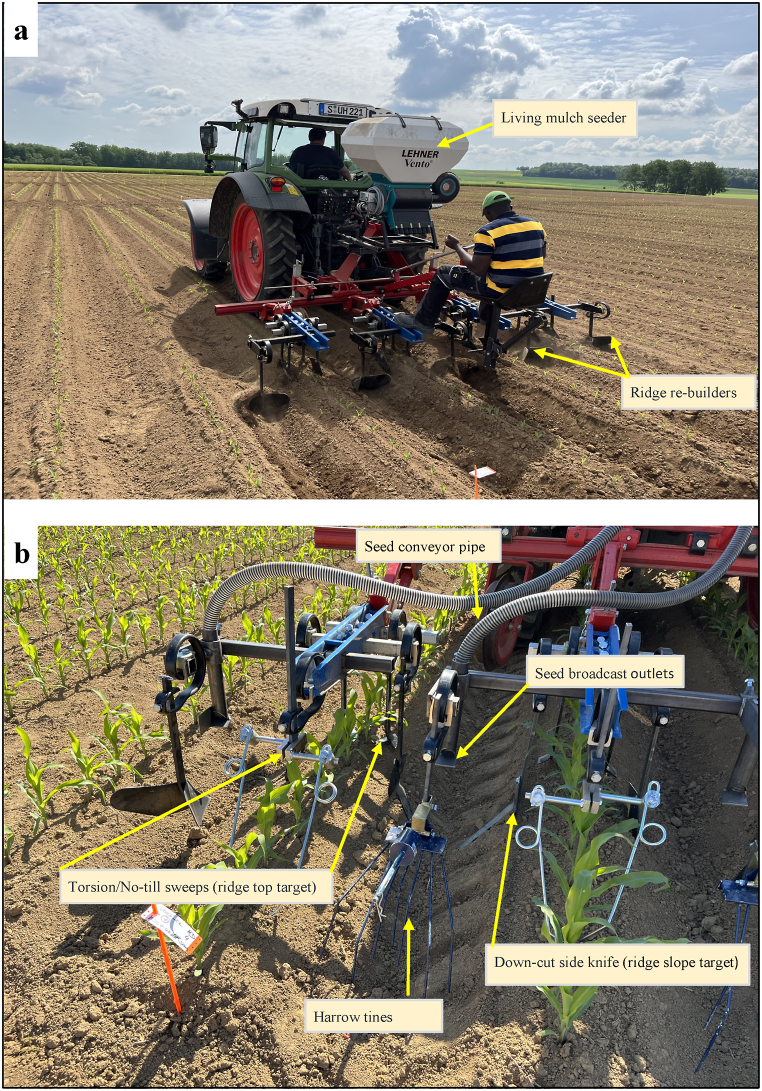
A combined ridge hoeing and living mulch seeding arrangement on a 3-m wide hoeing frame in 2022 (a). On ridge tops (intra-row target), pairs of no-till sweeps (8 cm wide) were positioned 5 cm away from maize plants. In the slopes of ridges (inter-row target), opposite pairs down-side-cut knives mounted 130° parallel to the slopes, followed by ridge re-builders positioned in the valleys (first pass of hoeing) with target seeding of living mulch (second pass of hoeing) in valleys (b).

In 2022, a combined hoeing and living mulch seeding prototype was developed (Fig. 3). Two pairs of no-till sweeps (8 cm wide) were mounted on ridge tops (5 cm away from maize plants to prevent crop damage) with a working depth of 4 cm. Eight no-till sweeps were required in total within intra-row areas (ridge tops). Bearing in mind the steepness of the ridge slopes, two opposite pairs of down-cut side-knives were bent 130° parallel to the slope with a working depth of 5 cm – such that opposite-facing pairs of down-cut side-knives formed a V-shape. This was followed by a V-shaped ridge re-building element to repair ridges consequent to minimal disruption of ridges from hoeing. Both elements (down-cut side-knives and ridge re-builders) allow for soil movement, uprooting, and burial of weeds at a relatively high tractor speed (5 km h^−1^). In total, four opposite pairs of down-cut-side knives and five ridge re-builders were mounted and precisely positioned within inter-row areas (slopes and valleys).

A living mulch seeder was built with a *Lehner Ventor®* seeder (Lehner Maschinenbau GmbH, 89198 Westerstetten, Germany) and integrated within the hoeing setup (Fig. 3). The seeder was calibrated to seed mixtures of 10 kg ha^−1^ of *Lolium perenne* L. and 20 kg ha^−1^ of *Trifolium subterraneum* L. at 660 g min^−1^ within 18 revolutions min^−1^ at 3 km h^−1^ driving speed. Five long, cylindrical-shaped pipes were attached to the seeding machine to convey and broadcast calibrated seeds precisely into the valley area (inter-row) at a steady air pressure. Seeds were broadcasted into the target area (valley) maintaining a 16 cm seeding height. A 30 cm wide harrow element was coupled behind each seed broadcasting element to further enhance the even spread and shallow burial of the seeds.

Before field treatments, all hoeing elements were adjusted in a test plot to ensure precise positioning (depth, distance, and angular alignments) within target areas, thereby ensuring minimal or no damage to the maize crop. Depending on the maize growth stages, hoeing elements were often readjusted to avoid crop damage. Hoeing and seeding elements were guided manually as the tractor automatically drives within an RTK-GNSS-built and seeded ridges at 5 km h^−1^. A manually guided hoe with three no-till sweeps (20 cm wide) per inter-row area (75 cm distance) was used to treat flat seedbeds (FT) in 2021 and 2022 with a driving speed of 5 km h^−1^.

### 2.2 Experimental site and design

The field experiment was conducted at the research station Ihinger Hof of the University of Hohenheim in 2021 and repeated in 2022. The research station is located in Southwest Germany, near Renningen (48°44′32.5″ N 8°55′31.1″ E) at an elevation of 475 m above sea level. The long-term average annual rainfall is 654 mm, and the long-term annual mean temperature is 7.9 °C. In both years, temperature were 2–3 °C warmer than the long-term average, but rainfall was almost equal to the long-term mean. The soil texture at Ihinger Hof is classified as loess loam with subsoil clay (Tschernosem-Parabrown soil). The agronomic details of the trials are shown in Table 1. Flat seedbeds (FT) were prepared with a *Fendt* 414 coupled with a 3 m rotary harrow at 5 km h^−1^ (8 cm deep) while new RTK-GNSS guided ridging elements (*Glühfosator®*, Frost Maschinenbau GmbH, 32469 Petershagen, Germany) was used to form re-compacted ridges. Maize (cv. Jackleen) was precisely sown with a *Monosem* seeder (Monosem inc. USA) on both RT and FT. Experiments were set up as a split-plot design. The design included tillage types (RT and FT) as the main plot and four weed control methods as a sub-plot factor.

**Table 1:** Experimental details including year, maize cultivar, preceding crop and soil tillage, sowing date (YYYY-MM-DD), seeding rate and row distance of both experiments at Ihinger Hof in 2021 and 2022.

Year	Crop, cv.	Preceding crop	Preceding soil tillage	Sowing date	Sowing rate (seeds m^−2^)	Row distance (m)
2021	Maize, cv. Jakleen	Winter Wheat	Ploughing (30 cm)	2021-05-25	8.8	0.75
2022	Maize, cv. Capuceen	Winter Wheat	Ploughing (30 cm)	2022-05-04	8.8	0.75

Tillage types (RT and FT) were randomly assigned to four blocks as main plots, and the four weed control methods were randomized within each tillage types (RT and FT) as sub-plots. Eight treatments were positioned randomly within each block with four repetitions. In total, 32 plots were realized. The weed control treatments consisted of a broadcast herbicide application, two hoeing passes, a single hoeing pass followed by precise seeding of living mulch within inter-row areas of FT and valleys of RT, and an untreated control (see Table 2). The weedy plots were left untreated for the entire growing season. However, it was ensured that the untreated control plots also received the same number of passes with the tractor wheels as the hoeing and herbicide treatments. The plot size in 2021 was 6 × 20 m and in 2022, it was 3 × 20 m, with the longer sides of each plot in the sowing or hoeing direction. Table 2 summarizes the weed treatments applied in both years with respective application timings.

**Table 2:** Overview of the treatments with application dates at Ihinger Hof in 2021 and 2022.

Treatment	2021	2022
Control	–	–
Herbicide a	Spectrum® Plus (2.5 L ha^−1^) + MaisTer® Power (1.0 L ha^−1^), 17-June	MaisTer® Power (1.0 L ha^−1^), 17-May
Twice hoeing	Two passes, 16-June and 27-June	Two passes, 17-May and 10-June
Hoe + LM	hoeing, 16-June + Living mulch b, 27-June	hoeing, 17-May + Living mulch, 02-June

a Spectrum® Plus = 212.5 g L^−1^ dimethenamid-P + 250 g L^−1^ pendimethalin, EC, BASF; MaisTer® power = 30.0 g L^−1^ foramsulfuron + 9.77 g L^−1^ thiencarbazone-Methyl + 0.85 g L^−1^ iodosulfuron + 15 g L^−1^ cyprosulfamide, SC, Bayer CropScience.

b *Lolium perenne* (manual seeded) in 2021, *Lolium perenne* L. + *Trifolium subterraneum* L. (machine seeded).

Treatments were performed at similar growth stages of the crop in both years. The first hoeing treatment was performed at crop growth stage BBCH 13, the second during BBCH 34, herbicide application in BBCH 13, and the living mulch was spread in BBCH 34 of the maize. Hoeing and living mulch seeding were performed with a driving speed of 5 km h^−1^. The herbicide application was carried out with a plot sprayer (Schachtner-Fahrzeug-und Gerätetechnik, Ludwigsburg, Germany) equipped with flat jet nozzles (Lechler, AD 120-02) and calibrated to deliver 200 L ha^−1^ at a pressure of 2.4 bar and a speed of 3.6 km h^−1^.

### 2.3 Data collection

#### 2.3.1 Weed/crop assessment

Weed species were identified, and weed densities were counted four times in each plot using a 0.1 m^2^ quadrat randomly placed within the two middle rows in each plot. Weed biomass was cut towards harvest and coverage were assessed visually using a 0.5 m^2^ frame, twice per plot. Weed control efficacy (WCE) was calculated according to Rasmussen [22] (see Equation (1)).

(1) WCE = 100 % - ds / (0.01 ∙ du) ………………………….

where ***ds*** is the weed density (weeds m^−2^) directly after application, and ***du*** is the weed density in the control treatment.

#### 2.3.2 Silage maize yield

In the 2022 experiment, two middle rows of 30 m^2^ in each plot were harvested using Kemper Häcksler® (Maschinenfabrik KEMPER GmbH & Co. KG, Stadtlohn, Germany) to determine fresh silage maize yield.

#### 2.3.3 Soil temperature determination

A double-sensor data logger (Tiny-Tag®, Gemini data loggers UK Ltd) was permanently installed on the top and valley areas of ridges while another single-sensor data logger (Tiny-Tag®, Gemini data loggers UK Ltd) was installed on FT beds immediately after maize was seeded to measure soil temperature. The sensor probes were plunged up to 15 cm soil depth. Soil temperature readings were automatically recorded at 20-min intervals from the maize sowing date until harvest in both years (2nd June – September 28, 2021, and 7th May – August 21, 2022).

#### 2.3.4 Soil moisture and root penetrability

To determine the impact of ridge re-compaction on soil moisture and root penetration, soil moisture and root penetrability tests were conducted simultaneously at 12, 36, 61, 77, and 96 days after sowing (DAS) in 2022. The percentage of soil moisture content was determined using the gravimetric method. Fifteen soil samples were taken randomly each in both RT and FT plots. Each sample was taken up to a depth of 30 cm with a 30 mm diameter soil auger. The soils were bulked together and 1000 g of soils were sub-sampled to oven dry for 48 h at 80 °C.

An *AGRETO*® soil compaction tester (AGRETO® Electronics GmbH, A-3820 Raabs, Austria) was used to determine root penetration. A 30° circular cone with an area of 129 mm^2^, and 12.8 mm diameter was selected for probing due to the firm soil texture in the site [23]. The depths of the plunge (cm) were recorded in correlation to the plunge resistance reading against 1.4 MPa (200 psi). Fifteen samples each were taken randomly within both RT and FT following ASABE standard procedure [24].

#### 2.3.5 Root biomass

To assess the influence of ridge re-compaction on root biomass, fresh roots of maize within 1 m^2^ were sampled in both FT and RT at 62 DAS and at harvest. Roots were dug 15–20 cm deep and uprooted with the stubble. The roots were washed with running water to remove heavy soil cleavage and then weighed.

### 2.4 Data analysis

The data were analyzed with SAS software 9.4 [25] as a split-plot design (Equation (2)). Before the two-way analysis of variance, the data were checked for homogeneity of variance and the assumption of normality. All visually-scored data were log-transformed to meet the normality assumption where necessary. Raw mean values were used for the graphical illustrations. Observed means were compared with LSD-test α ≤ 0.05. The following model was applied (see Equation (2))

(2) *y*_*ijh*_ = μ + *b*_*h*_ + α_*i*_ + β_*j*_ + (αβ)_*ij*_ + *f*_*ih*_ + *e*_*ijh*_ ……….

where *y*_*ijh*_ is the yield response of maize to *i*th tillage and *j*th weed control treatment within *h*th block, *μ* is the general mean (intercept), *b*_*h*_ is the effect of maize yield attributed to *h*th blocking, α_*i*_ is the yield effect attributed to *i*th tillage, β_*j*_ is the yield effect attributed to *j*th weed control, (αβ)_*ij*_ is the interaction effect between *i*th tillage and *j*th weed control*,* while *f*_*ih*_ is the error term of the main plot for block *h* with tillage-level *i;* and *e*_*ijh*_ is the general error term of the subplot in block *h* with *i*th tillage and *j*th weed control levels.

## 3 Results

### 3.1 Weather data

Table 3 shows the weather conditions at Ihinger Hof station between April and October of 2021 and 2022. The average monthly temperature and rainfall received ranged from 6 to 18 °C and 21–122 mm, respectively, in 2021, and from 7 to 21 °C and 24–99 mm in 2022. Total rainfall was higher in 2021 than in 2022, whereas the temperatures for both years were in a similar range.

**Table 3:** Weather conditions during the maize growing seasons in 2021 and 2022 (April–October); average monthly temperatures and monthly rainfall.

Months	Temperature (° C)		Rainfall (mm)	
	2021	2022	2021	2022
April	5.8	7.54	31.4	91.4
May	9.7	14.4	63.6	24.2
June	18.1	18.7	110.9	85.2
July	17.3	19.9	121.7	23.7
August	15.9	20.6	95	24.2
September	14.8	13.4	21.1	99.4
October	8.7	13.3	33.8	77.7

	Mean average		Total rainfall	
	**12.8**	**15.4**	**477.5**	**425.8**

### 3.2 Weed control assessments

#### 3.2.1 Weed flora composition

Experimental trials in 2021 and 2022 were characterized by different weed communities depending on tillage types (RT or FT). In 2021, weed flora was mainly composed of broadleaved weeds such as *Stellaria media* (L.) Vill. (22 % RT, 7 % FT), *Capsella bursa-pastoris* (L.) Medik. (20 % RT, 36 % FT), and *Lamium purpureum* L. (21 % RT, 4 % FT), as well as grasses (9 % RT and 29 % FT). In 2022, the most common weed species were *Fallopia convolvulus* L. (37 % RT, 24 % FT), and *Equisetum arvense* L. (53 % RT, 55 % FT).

#### 3.2.2 Influence of tillage and weed control treatments on response variables

The analysis of variance results in Table 4 show that there were no significant interactions between tillage and weed control treatments for all variables observed. Additionally, the impact of tillage on weed coverage, weed density, and weed dry weight was non-significant, except for weed density at 12 DAS and weed coverage at 96 DAS in 2022, with higher values for FT compared to RT (Table 4).

**Table 4:** Effect of tillage and weed control treatments and their interactions on weed density, weed coverage, and weed dry weight at 12, 36, and 96 days after sowing (DAS) in 2021 and 2022. Means with different letters within the same column indicate significant differences between treatments according to the LSD probability test (α ≤ 0.05). Abbreviated weed control treatments are defined as; Herbicide = broadcast herbicide application, Hoeing = two hoeing passes at 12 and 36 DAS, Hoe + LM = a single hoeing treatment combined with living mulch. Flat = Flat tillage (FT), Ridge = Ridge Tillage (RT).

**Treatment**		**Weed coverage (%)**						**Weed density (plants m**^**−**^**^2^)**						**Weed dry weight (g m**^**−**^**^2^)**	
		12 DAS		36 DAS		96 DAS		12 DAS		36 DAS		96 DAS		96 DAS	
		2021	2022	2021	2022	2021	2022	2021	2022	2021	2022	2021	2022	2021	2022
Tillage (T)	Flat	1.8b	5.3a	7.4a	36.1a	20.3a	59.8a	13.1a	36.4a	27.4a	77.9a	38.9a	41.1a	31.5a	416.2a
	Ridge	10.7a	5.4a	13.2a	28.4a	29.8a	43.3b	30.8a	23.8b	14.9a	68.2a	35.7a	29.9a	25.9a	295.2a
	SEM	2.1	0.7	3.2	6.4	7.4	7.3	8.8	5.7	10.7	19.9	12.6	9.7	10.5	66.3
Weed Control (WC)	Herbicide	9.6a	2.8b	0.9c	7.8c	0.7c	18.5c	33.0a	12.9b	0.6b	29.9b	3.4c	10.7b	0.1b	145.5b
	Twice hoeing	1.4b	2.1b	4.6bc	20.4b	13.9bc	42.6b	3.2b	21.3b	4.2b	45.4b	24.3bc	30.8b	19.2b	336.6b
	Hoe + LM	6.8 ab	2.0b	10.9b	30.6b	28.5b	57.4b	25.1 ab	23.2b	14.5b	78.9 ab	54.4 ab	37.9 ab	19.7b	292.5b
	Control	7.1 ab	14.6a	24.9a	70.1a	56.9a	88.7a	26.5a	63.1a	65.6a	138.3a	66.9a	63.0a	75.9a	648.1a
	SEM	2.1	0.6	3.2	6.1	7.0	7.0	8.2	5.9	10.7	20.5	12.6	10.0	10.6	75.3
ANOVA	T	<0.001	NS	0.09	NS	NS	0.03	NS	NS	NS	NS	NS	NS	NS	NS
	WC	0.05	<0.001	<0.001	<0.001	0.001	<0.001	0.07	<0.001	<0.001	<0.001	0.01	0.01	<0.001	<0.001
	T∗WC	NS	NS	NS	NS	NS	NS	NS	NS	NS	NS	NS	NS	NS	NS

SEM - Standard error of means.

NS - Non - significant.

##### 3.2.2.1 Weed coverage

In 2021, twice hoeing displayed 5 % and 14 % weed coverage at 36 and 96 DAS, respectively. Weed coverage at 36 and 96 DAS in the hoeing + living mulch seeded (Hoe + LM) treatment did not differ significantly to twice hoeing but was significantly different from the herbicide treatment.

In the 2022 experiment (Table 4), weed coverage varied significantly depending on the tillage at 96 DAS. FT recorded the highest weed cover (60 %) compared to RT (43 %). At 36 DAS, weed coverage for herbicide, twice hoeing, and Hoe + LM was statistically similar. At 96 DAS, weed coverage for twice hoeing and Hoe + LM did not differ significantly. However, both treatments were significantly different from the herbicide treatment.

##### 3.2.2.2 Weed density

In 2021, twice hoeing resulted in 94 % and 64 % reduction in weed density at 36 and 96 DAS, respectively, compared with the untreated control. Similarly, twice hoeing in 2022 resulted in 69 % and 53 % reduction in weed density at 36 and 96 DAS respectively. At 36 and 96 DAS, the weed density of twice hoeing was statistically equal to the herbicide treatment in both years (Table 4).

In 2022, the treatment with once hoeing and living mulch (Hoe + LM) reduced weed density at 36 and 96 DAS by 78 % and 19 %, respectively. However, weed density at 96 DAS (54 weed m^−2^) was not statistically different from weed density in the untreated control (67 weed m^−2^). Similarly, in 2022, Hoe + LM reduced weed density at 36 and 96 DAS by 42 % and 39 % respectively. Statistically, weed density for Hoe + LM (80 and 39 weed m^−2^) was not significantly different from untreated control (138 and 63 weeds m^−2^) at 36 and 96 DAS respectively (Table 4).

##### 3.2.2.3 Weed dry weight

In general, weed dry weight in the control plots was higher in the 2022 experimental year (648 g m^−2^) than in 2021 (76 g m^−2^). Herbicides resulted in a 99 % and 78 % reduction in weed dry weight in 2021 and 2022, respectively. In both experimental years, weed dry weight for twice hoeing and Hoe + LM decreased by 50–75 % compared to the untreated control. The reduction in dry weight for both treatments (twice hoeing and Hoe + LM) was equal to the herbicide in the two years of the study (Table 4). The same trends were observed in the performance of weed control treatments on weed dry weight within RT and FT (Table 5).

**Table 5:** Effect of weed control treatments on weed dry weight (g m^−2^)**,** weed control efficacy (%) at 36 DAS and silage maize yield (t ha^−1^) at 96 DAS within ridge and flat tillage systems in 2021 and 2022. Means with different letters within the same column indicate significant differences between treatments according to the LSD probability test (α ≤ 0.05). Abbreviated weed control treatments are defined as; Herbicide = broadcast herbicide application, Hoeing = two hoeing passes at 12 and 36 DAS, Hoe + LM = a single hoeing treatment combined with living mulch. Flat = Flat tillage (FT), Ridge = Ridge Tillage (RT).

Weed control treatments	Weed dry weight				Weed control efficacy				Silage maize yield	
	(g m^−2^)				(%)				(t ha^−1^)	
	2021		2022		2021		2022		2022	
	Flat	Ridge	Flat	Ridge	Flat	Ridge	Flat	Ridge	Flat	Ridge
Twice hoeing	29.0b	9.4b	452.7bc	198.8cd	94.2 ab	72.4c	58.4a	83.3a	35.1 ab	34.1b
Hoe + LM	22.2b	17.3b	276.4cd	336.1bcd	78.7bc	71.5c	64.7a	74.3a	35.1 ab	34.1b
Herbicide	0.04b	0.1b	183.7d	107.2d	98.8a	99.3a	81.2a	69.2a	35.5 ab	37.2a
Control	74.7a	76.9a	751.8a	544.4 ab	–	–	–	–	31.28c	31.55c

SEM	15.1		95.0		7.0		12.1		2.1	

SEM – Standard error of means.

### 3.3 Weed control efficacy

In 2021, RT resulted in lower weed control efficacy (WCE) than FT. For the herbicide treatment, WCE was 100 %, which was significantly higher than twice hoeing and Hoe + LM (71 % and 70 %, respectively) treatments. For FT, WCE of the herbicide treatment was 99 %, for twice hoeing 94 % and significantly lower than Hoe + LM with only 79 % (Table 5).

In 2022, within RT and FT, WCE between twice hoeing, Hoe + LM, and herbicide treatments did not differ significantly. However, within RT, a WCE of 80 %, 72 %, and 68 % was observed for twice hoeing, Hoe + LM, and herbicide treatments, respectively. Additionally, within FT, a WCE of 58 %, 64 %, and 82 % was observed for twice hoeing, Hoe + LM, and herbicide treatments, respectively (Table 5).

### 3.4 Silage maize yield

The influence of weed control treatments on maize biomass is shown in Table 5. Analysis of variance results show non-significant effects of tillage types and interaction of tillage and weed control treatments. Herbicide treatment within RT resulted in the highest maize yield (37.2 t ha^−1^). However, there was no significant difference between herbicide treatments in RT and FT. Within RT, maize biomass was reduced by 3 t ha^−1^ in the twice hoeing and hoe + LM treatments when compared with the herbicide treatment. However, both treatments (twice hoeing and hoe + LM) showed similar maize yield also for FT (Table 5).

### 3.5 Soil physical properties

**Fig. 4:**
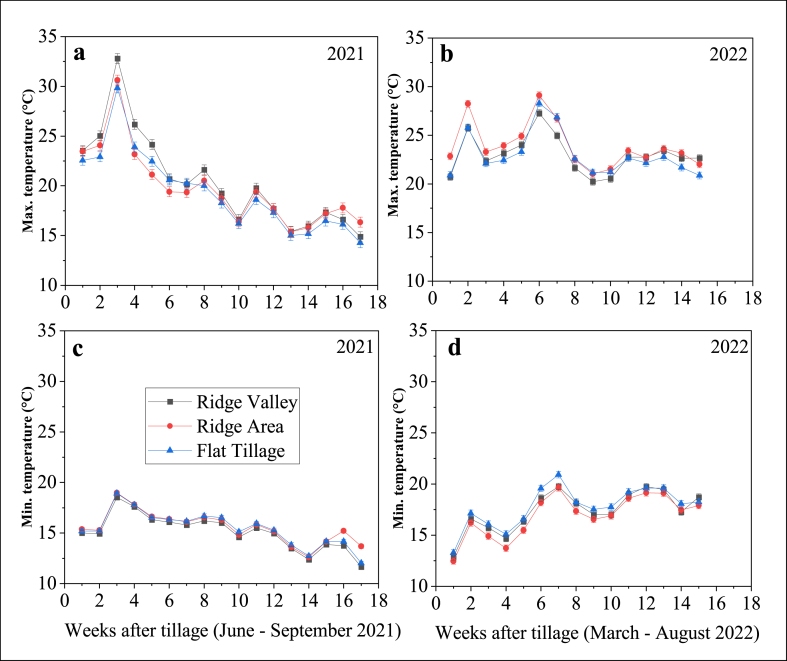
Weekly maximum (a and b), and minimum (c and d) soil temperature readings between June–September 2021 and March–August 2022 respectively within ridge area (red), ridge valley (black), and flat tillage (blue). Weekly temperature was averaged from readings taken at 20 min intervals. Error bars indicate differences between ridge area, ridge valleys and flat tillage based on Fischer's LSD test (p ≤ 0.05).

The minimum, average, and maximum soil temperature readings at 20-min intervals from 2nd June – September 28, 2021 and from 7th May – August 21, 2022 were averaged into weekly intervals and are presented in Fig. 4. Generally, average soil temperature in both years did not differ significantly between the ridge area, ridge valley, and flat tillage. However, significant minimum (night) and maximum (day) soil temperature margins were observed in certain weeks after sowing (WAS) in both years (Fig. 4a, b, 4c and, 4d).

In 2021, maximum soil temperature ranged from 15.4 to 30.6 °C, 14.8–32.8 °C and, 14.3–29.8 °C within ridge area, ridge valley, and flat tillage, respectively (Fig. 4a), while minimum soil temperature ranged from 12.6 to 18.9 °C, 11.6–18.5 °C and 12.0–18.9 °C within ridge area, ridge valley, and flat tillage, respectively (Fig. 4c). Across weekly periods, maximum soil temperature was significantly higher at 2, 3, 4, 5, and 8 WAS within the ridge valley (25.0, 32.7, 26.2, 24.1, and 21.6 °C, respectively) compared with similar soil temperatures within the ridge area (24.1, 30.6, 23.2, 21.1, and 20.5 °C, respectively) and flat tillage (22.9, 29.8, 23.9, 22.5, and 20.0 °C, respectively). In the last two WAS (weeks 16 and 17), maximum soil temperature shifted from the ridge valley to the ridge area (17.8 and 16.3 °C, respectively) compared with ridge valley (16.6 and, 14.9 °C, respectively) and flat tillage (16.1 and 14.3 °C, respectively) temperatures (Fig. 4a). Minimum soil temperatures were not significantly different in the three areas observed. However, the lowest soil temperature value was consistent within the ridge valley across the weeks observed (Fig. 4c).

In 2022, maximum soil temperature ranged from 21.0 to 29.1 °C, 20.2–27.3 °C and, 20.9–28.2 °C within the ridge area, ridge valley, and flat tillage, respectively (Fig. 4b), while minimum soil temperature ranged from 12.5 to 19.6 °C, 12.9–19.7 °C and, 13.3–20.9 °C within the ridge area, ridge valley, and flat tillage, respectively (Fig. 4d). Across weekly periods, maximum soil temperatures was significantly higher at 1, 2, 3, 4, 5, and 6 WAS within the ridge area (22.8, 28.2, 23.3, 23.9, 24.9, and 29.1 °C, respectively) compared with similar soil temperatures within ridge valley (20.7, 25.8, 22.4, 23.1, 23.9, and 27.3 °C, respectively) and, flat tillage (20.9, 25.8, 22.1, 23.2, 23.3, and 28.3 °C, respectively). At weeks 12, 13, 14, and 15, maximum soil temperatures within the ridge area (22.7, 23.6, 23.2, and 22.0 °C, respectively) and ridge valley (22.7, 23.4, 22.6, and 22.7 °C, respectively) were significantly higher compared with flat tillage temperatures (22.1, 22.8, 21.7, and 20.9 °C, respectively) (Fig. 4b). Minimum soil temperatures were not significantly different in the three areas observed except for the ridge area at weeks 9–11. However, the lowest soil temperature values were consistently lower within the ridge areas across the weekly periods (Fig. 4d).

Generally, irrespective of soil moisture conditions in both years, the highest maximum or minimum soil temperature values were consistent within either the ridge areas and ridge valleys across the weekly periods (Fig. 4).

**Fig. 5:**
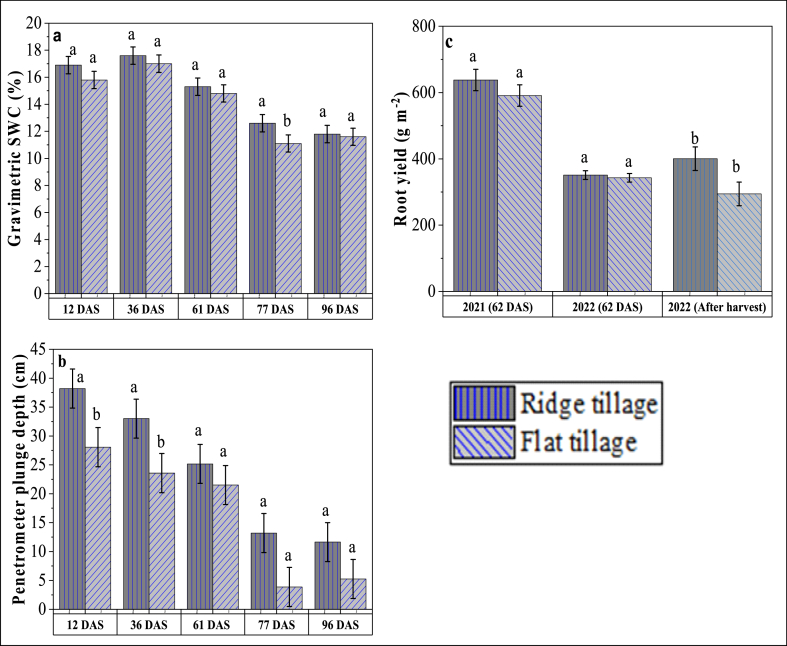
Influence of ridge re-compaction on gravimetric soil water content (SWC) (a), root penetration (b), and fresh root yield (c) of ridge and flat tillage across sampling periods. DAS – Days after sowing. Differing letters between tillage types within each period indicate significant differences according to the LSD test (p ≤ 0.05). Bars represent the standard error of the mean.

Soil moisture within the sampling periods ranged from 11 to 17.5 % with the highest moisture recorded at 12 and 36 DAS. However, soil moisture was non-significant between RT and FT (except 77 DAS where the lowest soil moisture was observed in FT under extremely dry soil conditions) (Fig. 5a).

Comparing penetrometer plunge depth against 1.4 MPa cone index (penetration resistance) between the RT and FT is sown in Fig. 5b. The results show that RT had a significantly higher plunge depth compared with FT. On average, RT had a 40 % higher plunge depth (7.8 cm) than FT.

The influence of re-compacted ridges on root biomass is shown in Fig. 5c. Although there was a slight increase in root biomass in RT compared to FT, differences in root biomass in both tillage types were non-significant at 60 DAS in both years. Root biomass sampled directly after harvest in 2022 recorded significantly higher weight gain in RT (106 g m^−2^) compared to FT. On average, a root yield gain of 53.9 g m^−2^ was observed in RT compared to FT.

## 4 Discussion

The new hoeing prototypes were successfully guided using RTK-GNSS-created ridges in the 2021 and 2022 experiments, with no record of physical crop damage due to hoeing in either years. In combination with hoeing, the precise seeding of living mulch in the valleys of ridges was successful. These results suggest the feasibility of precise post-emergent hoeing and broadcast seeding using RTK-GNSS-created ridges as a guide. Compared to expensive camera-guided hoeing systems [[26], [27], [28]], the developed hoeing prototypes could serve as a low-cost robotic weeding alternative, since cameras are not needed.

Weed densities were higher in RT than in FT. This supports earlier findings that RT exhibits higher weed pressure than FT [14,15]. Furthermore, in this study, broadleaved species such as *S. media*, *L. purpureum,* and *F. convolvulus* were more abundant within RT than grasses. The prevalence of broadleaved species within ridges in this study also agrees with the weed communities observed within ridge-cultivated maize and winter cereals [16,29], maize-soybean rotation [30], and organic vegetables [15,31].

The non-significant interaction of tillage and weed control implies that RT and FT did not influence weed control treatments. Twice hoeing at 12 and 36 DAS for both years of the study resulted in lower weed coverage, density, and dry weight, which was comparable to broadcast herbicide (71–80 % and 58–94 % WCE in RT and FT, respectively). This implies that twice hoeing passes on ridges with the new hoeing prototypes can substitute manual weeding or broadcast spraying of herbicides in RT systems. Also, mechanical hoeing treatment on ridges can reduce the manual weeding timing and operation cost [15]. Moreover, herbicide-resistant weed populations [16,17] within herbicide-dependent RT systems can be reduced. Notwithstanding, the hoeing treatments had a non-significant influence on silage maize yield in 2022 when compared with similar treatment in FT. Two or more trials may be necessary to establish the effect of hoeing and re-compaction on silage maize yield.

Soil physical properties were partly better in RT than in FT. Compared to FT across weeks, average weekly temperature rise of 0.3–2.4 °C and 0.2–2.1 °C was observed within ridge valleys in 2021 and ridge area in 2022 respectively, except for a few weeks where such pattern was not consistent. The temperature within ridges was slightly higher during the day and lower at night in 2022. This result is in agreement with Benjamin et al. [32] findings that ridges with a height ≥20 cm heat deeper and/or warmer than flat seedbeds due to large temperature gradients. Such persistent temperature gradients could probably have influenced selective germination of weed seeds on ridges as well as early crop vigor needed for competition against weeds. The high plunge depth in RT implies better crop root growth and development compared with flat seedbeds. This was also reported by Zhang et al. [12], Shi et al. [3], and Hu et al. [13]. The lower penetration resistance within RT suggest that more pore spaces for aeration and water intake were realized despite ridge re-compaction compared with FT. The latter might have influenced relative gains in root biomass. RT increased the number of nodal roots in maize compared with FT, which was related to lower root penetration resistance [33]. Soil moisture observed within RT and FT at different growth periods was mostly equal. However, RT retained higher soil moisture under extreme drought compared to FT in this study. The latter agrees with earlier studies that RT has the potential to retain optimal soil moisture under extreme drought or flooded conditions [7,8,10], particularly in temperate zones. However, studies on the impact of ridge re-compaction on soil physical properties on the long-term are needed to further support the findings in this study.

This study demonstrates that RT with re-compacted ridges in combination with the RTK-GNSS guiding system can be a sustainable new cropping practice.

## 5 Conclusion

This study underlines the potential of RT in conservation agricultural systems. It highlights the benefits of RT compared to FT providing better growing conditions for maize on the top of the ridges due to increased temperature gradients, lower root penetration resistance and better soil moisture conditions. The disadvantages of RT systems in regard to weed competition and weed control efficacy, especially when no herbicides are used, could be compensated by a new prototype of RTK-GNSS-guided ridging and an automatically guided hoeing system for mechanical weed control on the ridges and living mulches in the valleys. Therefore, we expect that RT will become more popular in conservation tillage systems.

## CRediT authorship contribution statement

**Oyebanji O. Alagbo:** Writing – original draft, Validation, Methodology, Investigation, Formal analysis, Data curation, Conceptualization. **Marcus Saile:** Writing – review & editing, Methodology, Conceptualization. **Michael Spaeth:** Writing – review & editing, Methodology. **Matthias Schumacher:** Writing – review & editing, Conceptualization. **Roland Gerhards:** Writing – review & editing, Visualization, Validation, Supervision, Software, Resources, Project administration, Methodology, Funding acquisition, Conceptualization.

## Data availability

Data associated with this study has not been deposited into any publicly repository. Data will be made available on request.

## Funding support

The project DiWenkLa (Digital Value Chains for a Sustainable Small-Scale Agriculture) is supported by funds of the 10.13039/501100005908Federal Ministry of Food and Agriculture (BMEL) based on a decision of the Parliament of the Federal Republic of Germany via the Federal Office for Agriculture and Food (BLE) under the innovation support program (grant reference 28DE106A18). DiWenkLa is also supported by the Ministry for Food, Rural Areas, and Consumer Protection Baden-Württemberg. Publishing fees supported by Funding Programme Open Access Publishing of University of Hohenheim.

## Declaration of competing interest

The authors declare that they have no known competing financial interests or personal relationships that could have appeared to influence the work reported in this paper.
